# A Comparison of Oxidative Lactate Metabolism in Traumatically Injured Brain and Control Brain

**DOI:** 10.1089/neu.2017.5459

**Published:** 2018-09-01

**Authors:** Ibrahim Jalloh, Adel Helmy, Duncan J. Howe, Richard J. Shannon, Peter Grice, Andrew Mason, Clare N. Gallagher, Michael P. Murphy, John D. Pickard, David K. Menon, T. Adrian Carpenter, Peter J. Hutchinson, Keri L.H. Carpenter

**Affiliations:** ^1^ Division of Neurosurgery, Department of Clinical Neurosciences, University of Cambridge, Cambridge, United Kingdom.; ^2^ Department of Chemistry, University of Cambridge, Cambridge, United Kingdom.; ^3^ Division of Neurosurgery, Department of Clinical Neurosciences, University of Calgary, Calgary, Ontario, Canada.; ^4^ MRC Mitochondrial Biology Unit, University of Cambridge, Cambridge, United Kingdom.; ^5^ Wolfson Brain Imaging Centre, Department of Clinical Neurosciences, University of Cambridge, Cambridge, United Kingdom.; ^6^ Division of Anaesthesia, Department of Medicine, University of Cambridge, Cambridge, United Kingdom.

**Keywords:** brain metabolism, microdialysis, NMR, 3-^13^C lactate, traumatic brain injury (human)

## Abstract

Metabolic abnormalities occur after traumatic brain injury (TBI). Glucose is conventionally regarded as the major energy substrate, although lactate can also be an energy source. We compared 3-^13^C lactate metabolism in TBI with “normal” control brain and muscle, measuring ^13^C-glutamine enrichment to assess tricarboxylic acid (TCA) cycle metabolism. Microdialysis catheters in brains of nine patients with severe TBI, five non-TBI brain surgical patients, and five resting muscle (non-TBI) patients were perfused (24 h in brain, 8 h in muscle) with 8 mmol/L sodium 3-^13^C lactate. Microdialysate analysis employed ISCUS and nuclear magnetic resonance. In TBI, with 3-^13^C lactate perfusion, microdialysate glucose concentration increased nonsignificantly (mean +11.9%, *p* = 0.463), with significant increases (*p* = 0.028) for lactate (+174%), pyruvate (+35.8%), and lactate/pyruvate ratio (+101.8%). Microdialysate ^13^C-glutamine fractional enrichments (median, interquartile range) were: for C4 5.1 (0–11.1) % in TBI and 5.7 (4.6–6.8) % in control brain, for C3 0 (0–5.0) % in TBI and 0 (0–0) % in control brain, and for C2 2.9 (0–5.7) % in TBI and 1.8 (0–3.4) % in control brain. ^13^C-enrichments were not statistically different between TBI and control brain, showing both metabolize 3-^13^C lactate via TCA cycle, in contrast to muscle. Several patients with TBI exhibited ^13^C-glutamine enrichment above the non-TBI control range, suggesting lactate oxidative metabolism as a TBI “emergency option.”

## Introduction

As an organ, the brain relies on glucose. Other potential energy metabolites such as lactate, other monocarboxylic acids, and ketone bodies are taken up into the brain under certain circumstances. Lactate, however, conventionally regarded as a waste product of “anaerobic” metabolism, can also act as an energy source, although its importance is debated.^1–4^ At the cellular level, however, there is an interchange of energy metabolites, and lactate is postulated to act in this capacity. The best described metabolic relationship between cells in the brain is the astrocyte-neuron lactate shuttle (ANLS) hypothesis.^4,5^

The human brain can metabolize lactate via the tricarboxylic acid (TCA) cycle, as shown by studies in animal^6–8^ and human brain (healthy volunteers and those with traumatic brain injury [TBI]).^9–11^ The study in human patients with TBI utilized microdialysis catheters to perfuse 3-^13^C lactate into the brain.^11^ Subsequent ^13^C nuclear magnetic resonance (NMR) analysis of returned microdialysates revealed ^13^C labeling in the TCA cycle derivatives glutamine and glutamate, thereby demonstrating that the human injured brain can metabolize lactate through the TCA cycle.^11^ The latter study was qualitative and did not quantify the degree of metabolism.

Boumezbeur and associates^9^ showed, in healthy human volunteers' brains, using intravenous ^13^C-lactate and *in vivo* ^13^C MRS of brain, that lactate is oxidatively metabolized to ^13^C-labeled glutamate/glutamine, hallmarks of TCA cycle operation. Glenn and colleagues^12^ performed an indirect study of brain metabolism, using arteriovenous measurements of ^13^CO_2_ as evidence of brain metabolism of ^13^C-lactate (given intravenously), in patients with TBI and healthy volunteers,^12^ but they did not undertake direct measurements of the brain.

Lactate shows evidence of being neuroprotective in injured brain.^13–20^ In particular, Bouzat and coworkers^16^ showed evidence for beneficial effects resulting from intravenous lactate administration in patients with TBI. There were significant increases in brain microdialysate lactate, pyruvate, and glucose. Reduction in brain microdialysate glutamate and a significant reduction in intracranial pressure (ICP) were also observed.^16^ The lactate solution administered was hypertonic, and it was not resolved how much of the apparent benefit on ICP was because of this property or because of the actual lactate itself.

Work by Ichai and colleagues^21^ in patients with TBI, however, showed that hyperosmolar sodium lactate solution (504 mmol/L; 1100 mosm/L) given intravenously was more effective at lowering ICP than a mannitol solution with an equivalent osmotic load (1160 mosm/L). Also, compared with saline (0.9%), a 0.5 mol/L sodium lactate solution was more effective at reducing the occurrence of raised ICP episodes in patients with TBI.^22^ Research by Quintard and associates^19^ showed that the beneficial effect of intravenous lactate on brain energetics (increasing brain extracellular glucose concentration) was a characteristic of those TBI patients with a high lactate/pyruvate ratio (>25) at baseline, rather than patients with baseline “normal” lactate/pyruvate ratio (<25).^19^

The human TBI brain's ability to oxidatively metabolize lactate versus normal, uninjured human brain has hitherto been unexplored directly. It is also unclear to what degree the TCA cycle in extracranial organs and tissues can metabolize lactate compared with the brain.

The aims of the present study were, therefore, to compare lactate metabolism in TBI with “normal” (non-TBI) control brain and resting muscle, and to quantify the production of glutamine isotopomers from lactate, to compare metabolism of lactate through the TCA cycle in these different tissues.

## Methods

### Patients

#### Ethics

The protocol for this study was approved by the National Research Ethics Service (NRES) Committee East of England–Cambridge Central (REC Reference No. 11/EE/0463). Informed consent was obtained from all participants in the control “normal” brain and muscle groups and assent from the relatives of those patients who had TBI. None of the patients in this study formed part of a previously published cohort. The study was performed in conformation with the spirit and the letter of the Declaration of Helsinki.

#### TBI brain microdialysis patients

Nine patients with TBI were studied. Patients with TBI were defined as those who had cranial trauma with consistent computed tomography scan findings and required invasive intracranial monitoring as part of their clinical management. Placements of microdialysis catheters in patients with TBI were into white matter not close to focal lesions. Patients were treated per local TBI-management protocols (including the following in all patients: endotracheal intubation, ventilation, sedation, and muscular paralysis) as in a previous study.^23^

#### Control “normal” non-TBI brain microdialysis patients

Five patients undergoing a craniotomy for the resection of a benign lesion whereby a microdialysis catheter could be placed safely into radiographically normal brain were selected as control subjects. The microdialysis catheter was placed via the craniotomy at the time of craniotomy under direct vision after resection of the benign lesion and tunneled under the scalp. The catheters were inserted at the edge of the craniotomy and directed away from the operative site into white matter; they came to lie approximately 2–3 cm away from the area of brain from which the benign lesion had been resected. The control patients were awake for the duration of microdialysis perfusion.

#### Muscle microdialysis patients

Five patients undergoing resections of acoustic neuromas that required thigh fat and fascia harvesting for dural closure were recruited for studying resting muscle. Microdialysis was performed as before, with patients under general anesthesia for the duration of microdialysis perfusion.^23^

### Materials

Central nervous system (CNS) perfusion fluid and T1 perfusion fluid, the compositions of which were described previously,^23^ were obtained from M Dialysis AB (Stockholm, Sweden). Sodium L-lactate (3-^13^C) (isotopic enrichment 99%, chemical purity 99%, microbiological and pyrogen tested MPT grade) 20% w/w in water was obtained from Cambridge Isotope Laboratories, Inc. (Tewksbury, MA) and was formulated to a final concentration of 8 mmol/L in CNS perfusion fluid (for brain) or T1 perfusion fluid (for muscle) by the Manufacturing Unit, Department of Pharmacy, Ipswich Hospital NHS Trust, who then tested the formulations to verify purity, sterility, freedom from endotoxins. and absence of pyrogenicity.

### Microdialysis technique

The microdialysis technique and catheters (M Dialysis 71) were the same as before.^23^ Catheters were placed into the right frontal white matter via a triple lumen cranial access device (Technicam, Newton Abbot, Devon, UK) in locations not close to focal lesions in the patients with TBI. Each patient received one microdialysis catheter, except for one patient with TBI in whom a left frontal catheter was placed via a craniotomy in addition to a right frontal catheter via cranial access device (Table 1). Catheters in the patients with TBI and the “normal” (non-TBI) brain control subjects were perfused with CNS perfusion fluid supplemented with sodium 3-^13^C lactate (8 mmol/L) at 0.3 μL/min. In resting muscle, catheters were perfused with T1 perfusion fluid supplemented with sodium 3-^13^C lactate (8 mmol/L) at 0.3 μL/min. Microdialysate collection vials from the patients with TBI were changed hourly and analyzed at the bedside using an ISCUS analyzer (M Dialysis AB) employing enzymatic colorimetric assays for glucose, lactate, pyruvate, and glutamate. In patients with TBI, brain tissue oxygen tension (PbtO_2_) data were collected using a Licox brain tissue oxygen sensor (GMS, Kiel-Mielkendorf, Germany) placed via the same cranial access device as the microdialysis catheter, and recorded at the bedside using ICM+ software (Cambridge Enterprise Ltd, University of Cambridge, UK).

**Table 1.** Traumatic Brain Injury Patient Demography

*TBI patient*	*Age*	*Sex*	*Injury mechanism*	*Initial injury severity: GCS at scene/15*	*Clinical outcome*	*Lactate perfusion period start time (hours from injury)*	*Catheter location (right frontal - RF; left craniotomy - LC)*	*PbtO_2_ during unsupple-mented period and during 3-^13^C lactate perfusion (mm Hg)*
TBI 1	51	M	Fall	E1 V2 M5	MDU/GRL	14	RF & LC (2 catheters)	–
TBI 2	36	M	RTC	E1 V1 M2	SDL^§^	34 & 60	RF	19.5, 29.3
TBI 3	17	M	RTC	E3 V4 M5	GRL	18	RF	–
TBI 4	48	M	Fall	E4 V4 M5	SDL	26	RF	–
TBI 5	45	M	Assault	–	Vg^*^	64	RF	–
TBI 6	22	F	RTC	E1 V2 M2	MDL	53	RF	–, 23.9
TBI 7	24	M	RTC	E1 V1 M5	MDU	83	RF	27.9, 26.0
TBI 8	47	M	RTC	E1 V2 M5	SDL	37	RF	37.4, 31.4
TBI 9	64	M	Fall	E3 V4 M6	D	98	RF	20.2, 35.1

M, male; F, female; RTC, road traffic collision; GCS, Glasgow Coma Scale score (E, eye opening; V, verbal response; M, motor response. Maximum total score = 15); PbtO_2_, brain tissue oxygen tension (median values), – not measured. In patients with RF catheter location, the microdialysis catheter and PbtO_2_ probe were inserted via a triple lumen cranial access device into white matter. The LC (left craniotomy) microdialysis catheter (traumatic brain injury [TBI] patient 1) was also in white matter. Clinical outcomes (categorized by Extended Glasgow Outcome Scale criteria): GRL, good recovery (lower); MDU, moderate disability (upper); MDL, moderate disability (lower); SDL, severe disability (lower); Vg, vegetative; D, dead. ^**§**^ Outcome on transfer to rehabilitation hospital. ^*^Outcome on discharge from NCCU. Other outcomes were from clinic.

### NMR analysis

Microdialysate sample preparation and NMR analysis were performed as before.^23^ Brain microdialysate samples were pooled into 24-h periods for each patient. For NMR analysis, 20 μL of deuterium oxide (D_2_O) and 50 μL internal reference standard from a stock solution of 24.0 mmol/L 3-(trimethylsilyl)-1-propanesulfonic acid sodium salt (also termed 2,2-dimethyl-2-silapentane-5-sulfonate sodium salt or 4,4-dimethyl-4-silapentane-1-sulfonate sodium salt; DSS) in CNS perfusion fluid were added to 180 μL of the pooled microdialysate sample. Muscle microdialysate samples were pooled into 8-h periods for each patient, these shorter periods (compared with 24-h pool for each brain patient) being because of clinical constraints. Then 20 μL of D_2_O and 70 μL from a stock solution of 2.84 mmol/L DSS were added to 120 μL of the pooled microdialysate sample. Pooled samples, after addition of D_2_O and DSS, were stored at −80°C. For NMR analysis, samples were transferred into 3 mm NMR tubes (Hilgenberg GmbH, Malsfeld, Germany). All chemical shifts were referenced to DSS = zero ppm (Hz per MHz) in the NMR spectra.

The ^13^C and ^1^H NMR spectra were acquired as described previously^23^ on a Bruker Avance III HD 500 MHz spectrometer (Bruker BioSpin GmbH, Karlsruhe, Germany) with a dual ^13^C/^1^H cryoprobe (CP DUL500C/H, Bruker BioSpin GmbH), and TopSpin software (Bruker GmbH). using the pulse programs noesypr1d and zgpg30 for ^1^H and ^13^C spectra, respectively.^23^ These are detailed Supplementary Methods (see online supplementary material at ftp.liebertpub.com). Because of the small sample size of the microdialysates, which necessitated long acquisition times to achieve adequate sensitivity, it was impracticable to run fully relaxed spectra without nuclear Overhauser enhancement. Therefore, we undertook detailed calibration of the signal intensities for each carbon position within the glutamine molecule (see paragraph below on quantification).

**FIG. 1.**
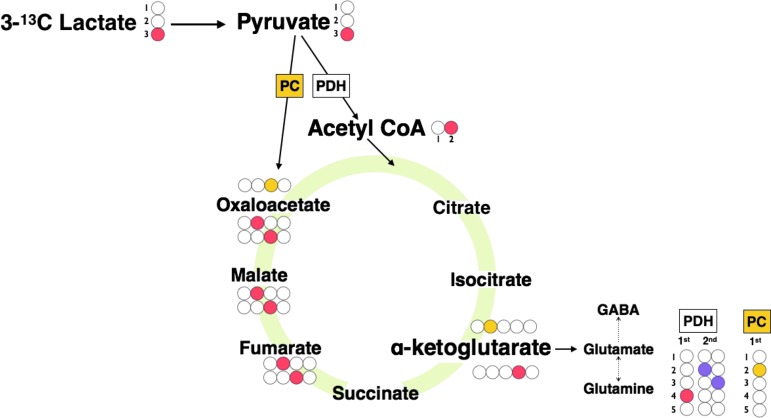
Schematic of ^13^C labeling patterns derived from 3-^13^C lactate. 3-^13^C lactate is metabolized to 3-^13^C pyruvate, which then enters the tricarboxylic acid (TCA) cycle via conversion to 2-^13^C acetyl CoA by pyruvate dehydrogenase (PDH) or via pyruvate carboxylase (PC) to 3-^13^C oxaloacetate. 2-^13^C acetyl CoA is metabolized to citrate, isocitrate, and then to 4-^13^C α-ketoglutarate where it can then spin out as 4-^13^C glutamate, which is then converted to 4-^13^C glutamine. Consequently 4-^13^C glutamine is produced on the first turn of the TCA cycle. If 4-^13^C α-ketoglutarate remains within the TCA cycle, it is metabolized to equal proportions of 2-^13^C malate and 3-^13^C malate because of “randomization” of label in the fumarate to malate step, because fumarate is a symmetrical molecule whereas malate is asymmetric. If malate continues within the TCA cycle, equal amounts of 2-^13^C glutamine and 3-^13^C glutamine are produced on the “second turn” of the TCA cycle. When 3-^13^C lactate is metabolized via PC to 3-^13^C oxaloacetate, 2-^13^C α-ketoglutarate is produced, which results in 2-^13^C glutamine produced on the “first turn” of the TCA cycle. For the labeling patterns of the second turn, we have assumed for schematic illustration that the incoming second acetyl-CoA molecule is not enriched with ^13^C. These patterns were consistent with our observations; see Results section and Supplementary Table 2. Colored filled circles indicate position of ^13^C atoms within molecules. Red indicates entry via PDH into the TCA cycle, first turn, and purple for second turn; yellow indicates entry via PC, first turn.

Interpretation of the NMR results was based on biosynthetic pathways summarized schematically in Figure 1, which shows the glutamine labeling patterns corresponding to TCA cycle metabolism of lactate. To summarize, 3-^13^C lactate is metabolized via pyruvate dehydrogenase (PDH) to 4-^13^C glutamine on the first turn of the TCA cycle and to equal amounts of 2-^13^C glutamine and 3-^13^C glutamine on the second turn of the TCA cycle. When 3-^13^C lactate is metabolized via pyruvate carboxylase (PC), 2-^13^C glutamine is produced on the first turn of the TCA cycle.

Quantification of glutamine isotopomers was performed with the same methodology used previously.^24^ Calibration curves were constructed using glutamine standards of varying concentration prepared in CNS perfusion fluid with the same fixed concentration of D_2_O and DSS used for the preparation of brain microdialysate samples and run under identical NMR conditions. The peak areas (measured using TopSpin software) of the glutamine ^13^C NMR signals in the microdialysate samples relative to the DSS internal standard were used with reference to the calibration curves to calculate ^13^C concentration. Notably, the ^13^C NMR signals of each of the glutamine carbon positions (C2, C3, C4) were individually calibrated, thereby taking into account the different strengths of nuclear Overhauser enhancement effect at these individual positions. Peak areas of the ^1^H NMR spectra fitted using Chenomx software (Chenomx Inc, Edmonton, Canada) were used to calculate ^12^C concentration.

Fractional enrichment (%) is defined as 100 × [^13^C] / ([^13^C] + [^12^C]) where square brackets indicate concentrations of the relevant species. [^13^C] was determined from the ^13^C NMR spectra and [^12^C] from the ^1^H spectra. Natural abundance of ^13^C is 1.1% of all carbon atoms, and ^13^C fractional enrichment values for glutamine were expressed after subtracting this naturally occurring ^13^C background from the ^13^C singlet signals.

### Statistical analysis

Statistical analyses were performed using SPSS21 for Mac (IBM SPSS Statistics, NY). Changes of ISCUS microdialysis parameters between baseline and 3-^13^C lactate perfusion periods were assessed for statistical significance with Wilcoxon signed rank test with a preselected *p* value of 0.05 for statistical significance. Student paired *t* test (StatView 5, SAS Institute, Cary, NC) was used additionally for corroboration. Mann-Whitney *U* test (on SPSS21) was used to compare glutamine fractional enrichment between TBI and normal brain microdialysates.

## Results

### Demography

Nine patients with severe TBI (eight male, one female) aged 22–64 years (median 45 years) were studied using 3-^13^C lactate (8 mmol/L) perfusion via the microdialysis catheter for 24 h. Eight of the nine patients had simultaneous collection of microdialysates for NMR analysis. One of these patients had 3-^13^C lactate perfusion via bilateral catheters: one catheter placed via a bolt (cranial access device) into right frontal white matter and the other catheter via craniotomy into left frontal white matter. Apart from this one craniotomy catheter, all other catheters were placed into the right frontal white matter via a bolt that was not close to focal lesions. Microdialysis technique was in accord with the 2014 Consensus Statement guidelines,^25^ so catheters were never placed in lesions, and the first hour of microdialysate collected was never used for clinical monitoring to eliminate any unreliable results from insertion trauma and the pump flush sequence.

One patient underwent two 24-h periods of 3-^13^C lactate perfusion. Thus, a total of 10 samples from eight patients with TBI underwent NMR analysis. Six of the nine patients with TBI also underwent an unsupplemented microdialysis period with plain CNS perfusion fluid (without 3-^13^C lactate). Demographic details of the TBI patients are presented in Table 1, which includes Glasgow Coma Scale (GCS) scores as a measure of initial severity of the traumatic injury, and details of catheter placement.

As controls for comparison with the TBI brain, microdialysis catheters were inserted in macroscopically normal-appearing brain (frontal white matter), using the same 3-^13^C lactate (8 mmol/L) perfusion at 0.3 μl/min, in five non-TBI patients (two male, three female) aged 42–79 years (median 69 years) undergoing surgery for benign brain tumors. Thigh (quadriceps, resting) muscle was studied similarly in five other patients (two male, three female) aged 59–68 years (median 61 years) undergoing surgery for acoustic neuroma resection. Muscle microdialysis was limited to periods of 8 h during which the patients were anesthetized undergoing acoustic neuroma surgery.

### Bedside analysis

**FIG. 2.**
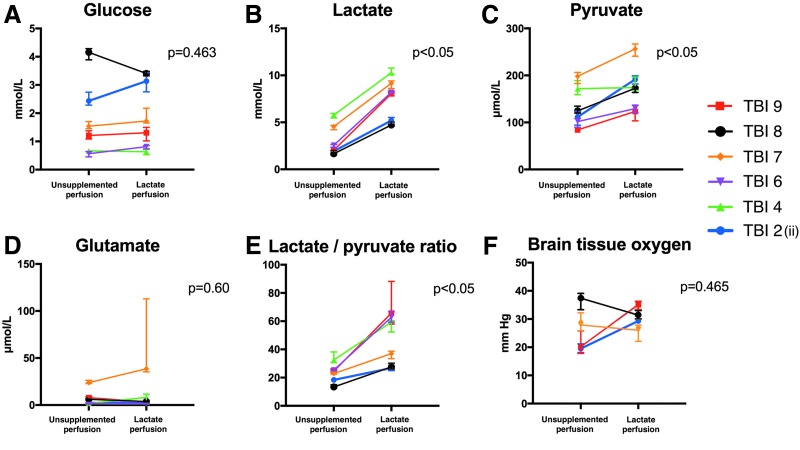
ISCUS microdialysate and brain tissue oxygen tension (PbtO_2_) data for unsupplemented perfusion period versus 3- ^13^C lactate (8 mmol/L) perfusion in patients with traumatic brain injury (TBI). (**A–E**) ISCUS clinical microdialysis analyzer measurements and (**F**) PbtO_2_ data: during a 24-h period with unsupplemented plain CNS perfusion fluid and during 24 h perfusion with 3-^13^C lactate (8 mmol/L). Error bars represent interquartile ranges. ISCUS data are presented numerically in Supplementary Table 1 and PbtO_2_ values in Table 1.

Unsupplemented microdialysis perfusion results were compared with the 3-^13^C lactate perfusion period in patients with TBI. Microdialysate measurements (ISCUS analyzer) of glucose, lactate, pyruvate, glutamate, and lactate/pyruvate ratio are shown in Figure 2 for six of the nine patients with TBI. The other three patients with TBI who underwent 3-^13^C lactate microdialysis did not undergo a period of unsupplemented perfusion fluid microdialysis. Median values and interquartile ranges (IQRs) are shown for a 24-h period with unsupplemented plain CNS perfusion fluid and for a 24-h period with 3-^13^C lactate perfusion (8 mmol/L).

As shown in Figure 2 (A-E), four of six patients demonstrated increases and two of six patients decreases in glucose between an unsupplemented microdialysis period (with plain CNS perfusion fluid) and the 3-^13^C lactate perfusion period with a mean difference of +11.9%. This increase was not significant (*p* = 0.463). As expected, 3-^13^C lactate perfusion resulted in a significant increase in microdialysate lactate (*p* = 0.028); all patients demonstrated increases in lactate between unsupplemented and 3-^13^C lactate perfusion periods with a mean difference of +174%. Pyruvate increased significantly between unsupplemented and 3-^13^C lactate perfusion period with a mean difference of 35.8% (p = 0.028). The lactate/pyruvate ratio was also observed to rise significantly with a mean difference of 101.8% (*p* = 0.028). Glutamate concentrations did not change significantly between unsupplemented and 3-^13^C lactate perfusion periods with a mean difference of 51.1% (*p* = 0.60).

These statistical comparisons were by Wilcoxon signed rank test (a paired nonparametric test). These results were corroborated by paired Student *t* test (a paired parametric test): glucose (nonsignificant, *p* = 0.72), lactate (significant, *p* = 0.0003), pyruvate (significant, *p* = 0.0108), lactate/pyruvate ratio (significant, *p* = 0.0078), and glutamate (nonsignificant, *p* = 0.44).

PbtO_2_ (Fig. 2F) did not change significantly between unsupplemented and 3-^13^C lactate perfusion periods, although complete data were only available for four patients, two of whom showed an increase and two a decline in PbtO_2_ during lactate perfusion compared with unsupplemented perfusion (Wilcoxon signed rank test, *p* = 0.47; paired Student *t* test *p* = 0.45).

### Metabolic products derived from 3-^13^C lactate

**FIG. 3.**
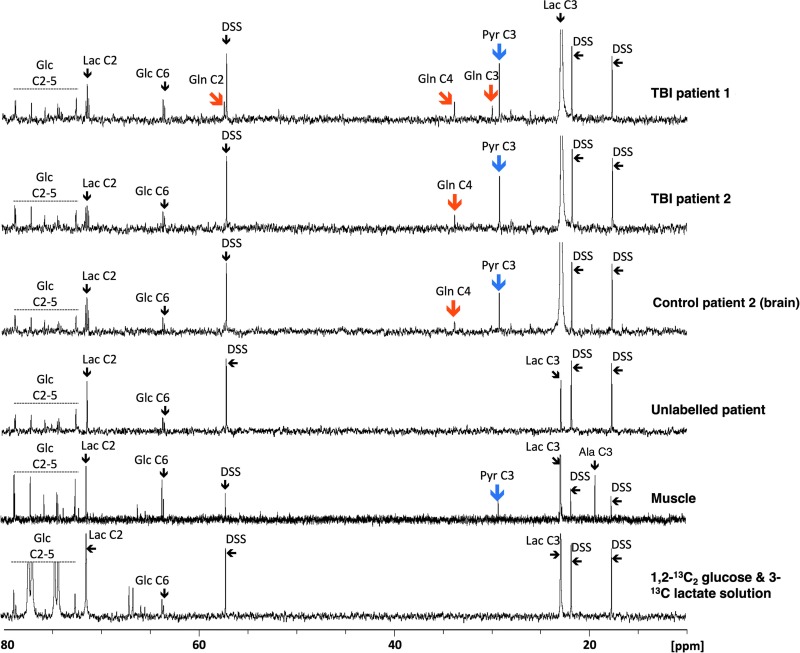
Illustrative examples of ^13^C nuclear magnetic resonance spectra for microdialysates from patients who received perfusion with 3-^13^C lactate. Traumatic brain injury (TBI) brain (uppermost two spectra, 24 h perfusion; patients TBI 1 and TBI 2, respectively), control (non-TBI) “normal” brain (third spectrum, 24 h perfusion; patient CB 2), and muscle (fifth spectrum, 8 h perfusion). An example of brain microdialysate from an unlabeled TBI patient with plain (unsupplemented) perfusion fluid is shown for comparison (fourth spectrum). Blue arrows indicate pyruvate C3 enrichment, and orange arrows indicate glutamine C4, C3, and C2 enrichment. DSS, 4,4-dimethyl-4-silapentane-1-sulfonate sodium salt (the internal reference standard); Glc, glucose; Gln, glutamine; Lac, lactate; Pyr, pyruvate, Ala, alanine. Spectra were run from −20 ppm to +250 ppm. The main reference DSS signal at 0 ppm is not shown in the range illustrated. All chemical shift values (ppm, on x-axis of spectrum) are relative to DSS at 0 ppm. The sixth spectrum is a standard solution consisting of a mixture of 1,2-^13^C_2_ glucose (2 mmol/L) and 3-^13^C lactate (4 mmol/L) in central nervous system perfusion fluid showing the signals produced by these substrates.

Illustrative examples of ^13^C NMR spectra of the microdialysates are shown in Figure 3. Resulting from 3-^13^C lactate perfusion, TBI brain, “normal” (non-TBI) control brain, and muscle microdialysates included clearly visible additional peaks, distinct from the lactate peaks. These additional peaks were not visible in microdialysates from patients receiving unsupplemented perfusion fluid (unlabeled patient in Fig. 3), indicating cellular uptake and metabolism of the 3-^13^C lactate. The substrate lactate C3 at 22.8 ppm produced the largest signal as expected.

A clearly visible singlet peak at 29.2 ppm for pyruvate C3, which was not visible in unlabeled TBI microdialysates, was present in TBI brain, “normal” non-TBI control brain, and muscle microdialysates as a result of 3-^13^C lactate perfusion. No other ^13^C pyruvate isotopomers were detected. In our muscle microdialysates, a ^13^C NMR signal at 19.3 ppm corresponding to alanine C3 was observed (Fig. 3), although we did not quantify enrichment. Identity of the alanine C3 was corroborated by ^1^H–^13^C heteronuclear single quantum correlation.

**FIG. 4.**
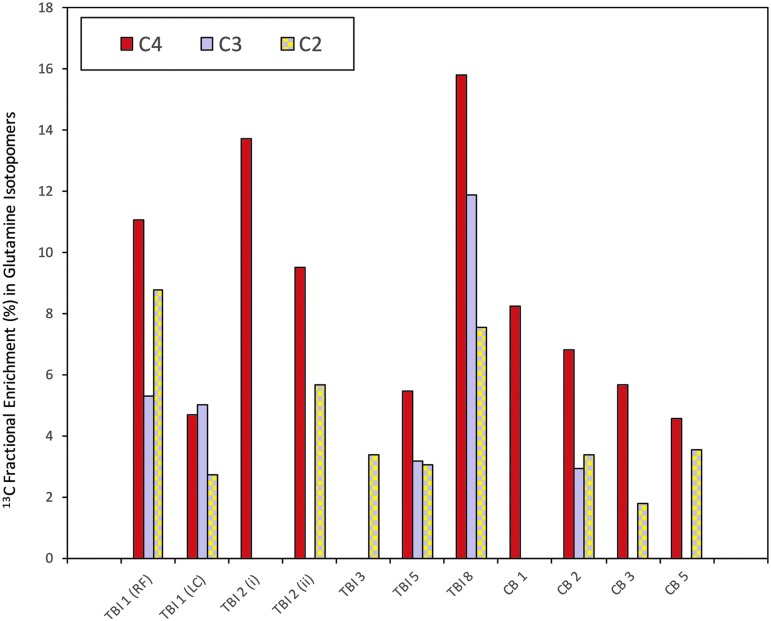
^13^C labeling of glutamine isotopomers. ^13^C fractional enrichment (%) values in the glutamine isotopomers C4, C3, and C2 (after subtraction of the 1.1% natural abundance background) in patients with traumatic brain injury (TBI) and control (non-TBI) “normal” brain patients. Four other patients (TBI 4, TBI 6, and TBI 7 and CB 4) showed no detectable ^13^C enrichment above natural abundance background, and therefore are not illustrated. RF, right frontal; LC, left craniotomy; CB, control brain; (i) and (ii), first and second periods of microdialysis, respectively. All the above ^13^C fractional enrichments in glutamine were in the form of single labeling. Double-labeling was not detected in glutamine. For further details, see Table 1 and Supplementary Table 2.

Labeling of glutamine derived from 3-^13^C lactate was detected in seven of 10 TBI and four of five “normal” (non-TBI) control brain microdialysates, but not in muscle. In the patients with TBI, glutamine labeling was detected in the C4 position (33.7 ppm) in six of 10 samples, in the C2 position (57.1 ppm) in six of 10 samples, and in the C3 position (30.0 ppm) in four of 10 samples. In “normal” non-TBI control brain microdialysates, C4 labeling was detected in four of five, C2 labeling in three of five, and C3 labeling in one patient. See Figure 4 for further details.

The greater fractional enrichment of glutamine C4 over C2 enrichment indicates that the entry of ^13^C to the TCA cycle was predominantly via PDH rather than PC. The reason is that when 3-^13^C lactate enters the TCA cycle via PDH, it produces labeling at C4 of glutamine on the first turn (and C2 and C3 glutamine isotopomers only on subsequent turns), whereas if 3-^13^C lactate enters the TCA cycle via PC, it leads to labeling on C2 of glutamine on the first turn of the TCA cycle (see Fig. 1). In only one of the patients (TBI patient 3) was C2 labeling greater than C4, and in this patient, C4 labeling was undetectable above background.

**FIG. 5.**
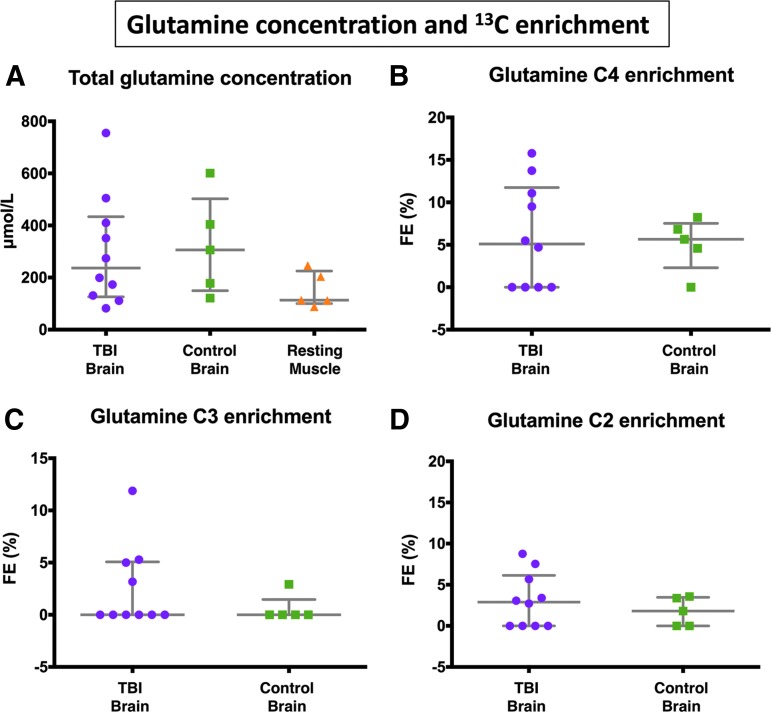
Microdialysate nuclear magnetic resonance (NMR) measurements of ^13^C labeling: glutamine enrichment. (**A**) Total glutamine concentration measured using ^1^H NMR, in TBI brain, control (non-TBI) brain, and muscle. (**B–D**) Results from microdialysis perfusion with 3-^13^C lactate (8 mmol/L) for 24 h in brain: TBI or control (non-TBI) “normal”. Although glutamine was detectable in muscle (measured using ^1^H NMR), ^13^C fractional enrichment of glutamine above natural abundance was not detectable in resting muscle. Natural abundance of ^13^C was assumed to be 1.1%, and ^13^C fractional enrichment values for glutamine were expressed after subtracting this naturally occurring ^13^C background. For patient identities, see Fig. 4 graph and legend. Data points depict individual measurements, and bars indicate medians and interquartile ranges.

Overall, there were no statistically significant differences between TBI and “normal” non-TBI control brain microdialysates for glutamine fractional enrichment values for C4, C3, or C2 isotopomers (Fig. 4, 5, and Supplementary Table 2; see online supplementary material at ftp.liebertpub.com). C4 glutamine fractional enrichment (median and IQR) was 5.1 (0–11.1) % in TBI and 5.7 (4.6–6.8) % in the non-TBI control brain. C3 enrichment was 0 (0–5.0) % in TBI and 0 (0–0) % in the non-TBI control brain. C2 enrichment was 2.9 (0–5.7) % in TBI and 1.8 (0–3.4) % in the non-TBI control brain. All of the above enrichments in glutamine were in the form of single labeling. Double-labeling was not detected in glutamine, evidenced by the absence of ^13^C NMR doublet signals (for further details, see footnote to Supplementary Table 2). No glutamine enrichment above that expected from natural abundance ^13^C was detected in muscle microdialysates.

## Discussion

In this study, 3-^13^C lactate microdialysis of human muscle and brain with subsequent ^13^C NMR analysis of microdialysates demonstrated TCA cycle metabolism of lactate to glutamine in the brain but not muscle, although muscle metabolized lactate to pyruvate. The TCA cycle metabolism of lactate produced similar amounts of glutamine in “normal” (non-TBI) control and injured brain. Inspecting the glutamine labeling pattern revealed that the principal entry of lactate to the TCA cycle was via PDH.

### Labeling of glutamine indicates operation of the TCA cycle

This study adds further evidence in support of lactate acting as an oxidative energy substrate in the human brain. 3-^13^C lactate administered to the brain by the microdialysis catheter resulted in the production of glutamine (labeled at the C4, C3, and C2 positions), which requires lactate to have been metabolized by the TCA cycle.

Detection of labeling in glutamine is a clear indicator of TCA cycle metabolism of the labeled substrate. The reason is that a portion of the TCA cycle intermediate α-ketoglutarate is converted into the spinout product glutamate, which is enzymatically interconvertible with glutamine that dominates extracellularly. The prevailing view of glutamate-glutamine cycling involves trafficking between astrocytes and neurons. Glutamate is produced *de novo* by the transamination of α-ketoglutarate, an intermediate of the TCA cycle. Glutamate released from neurons during synaptic transmission is taken up by astrocytes and converted to glutamine by the action of glutamine synthetase, which is present predominantly in astrocytes. Glutamine is released by astrocytes into the interstitial extracellular fluid, where it is imported by neurons and converted to glutamate by phosphate-activated glutaminase. These processes are known as the glutamate-glutamine cycle.^26–30^ Consequently, carbons released from neurons during the synaptic release of glutamate are thus recycled to neurons in the form of glutamine from astrocytes. Also, astrocytes/glia can themselves synthesise glutamate *de novo* via the TCA cycle α-ketoglutarate (see above) and the ensuing glutamate can be converted to glutamine by the same astrocytes/glia.^31,32^

In summary, glutamine is derived from the TCA cycle either from the transamination of α-ketoglutarate to produce glutamate, followed by the action of glutamine synthetase in astrocytes, or following the capture by astrocytes of leftover glutamate after neurotransmission. Glutamate uptake by astrocytes is essential to terminate its effect as a neurotransmitter and to prevent extracellular glutamate from reaching excitotoxic levels.^5^ The present study indicates TCA cycle metabolism of 3-^13^C lactate, because ^13^C signals for glutamine (C4, C3, and C2) suggest that the TCA cycle is operating in the regions of interest of the catheters, because glutamine is a product of glutamate that is a spinout of the TCA cycle.

In muscle, uptake and metabolism of 3-^13^C lactate was evident by the generation of 3-^13^C pyruvate and 3-^13^C alanine (Fig. 3). This is consistent with literature reports that muscle can convert lactate to pyruvate (by the action of lactate dehydrogenase), which is then transaminated (by alanine aminotransferase) to form alanine,^33^ so lactate C3 becomes pyruvate C3, which then becomes alanine C3, and the process is reversible. Further downstream, metabolites of 3-^13^C pyruvate in the form of TCA cycle intermediates were not detected in muscle, in our study. The likelihood is that glutamine does not function as an intercellular metabolite in resting muscle in the same way as it does in brain. The fact that 3-^13^C lactate contributed to an extracellular pool of pyruvate, however, indicates the activity of lactate dehydrogenase in metabolizing lactate to pyruvate. This suggests contribution of lactate to an intracellular pool of pyruvate that could potentially undergo mitochondrial oxidative metabolism.

In resting muscle, however, we found no evidence to demonstrate whether lactate contributes significantly to oxidative metabolism. This may have been at least partly because of the shorter periods that muscle underwent 3-^13^C lactate microdialysis (8 h, because of clinical constraints), compared with brain 3-^13^C lactate microdialysis (24 h) and to the fact that this muscle was resting. In contrast, there is literature evidence that exercising muscle not only produces lactate but also consumes and oxidizes lactate.^34,35^ Because of clinical limitations, we were unable to compare exercising muscle with resting muscle in our own study.

### TCA cycle metabolism of lactate occurs in both TBI and non-TBI control brain

This quantitative study supports a previous ^13^C-labeling microdialysis study that demonstrated qualitatively the metabolism of lactate via the TCA cycle in the injured human brain.^11^ In the present study, we have quantified labeling (% enrichment) and demonstrated that TCA cycle metabolism of lactate is not limited to the injured brain but also occurs to a similar median degree in the “normal” (non-TBI) control brain. In the present study, no statistically significant differences between TBI and non-TBI control brain were observed for ^13^C enrichment values for any of the glutamine isotopomers. Nevertheless, the upper range values for ^13^C enrichment in glutamine were higher in TBI than non-TBI control brain (see Fig. 5), which concur with the idea that in certain cases, lactate oxidative metabolism via the TCA cycle is an “emergency option” for injured brain. Several literature reports in other studies support this idea.^36,37^ Our results are also compatible with the ANLS hypothesis,^4,5^ although, of course, the lactate supply was exogenous in our study, and our technique *per se* does not distinguish the cell-type(s) metabolizing the 3-^13^C lactate.

While lactate is often regarded as a neuronal substrate,^4^ astrocytes have also been shown to oxidatively metabolize lactate via the TCA cycle,^38^ albeit to a lower degree than neurons. Competition studies *in vitro,* employing 1-^13^C glucose (with unlabeled lactate) and 3-^13^C lactate (with unlabelled glucose) in neuronal cultures demonstrated that lactate was preferentially consumed by the neurons for their oxidative metabolism.^39^ *In vivo,* perfused ^13^C-lactate was considered as mainly a neuronal substrate in both animals and humans (see Introduction). These results are supported by the work of Mächler and coworkers,^40^ using the Laconic probe, showing a lactate gradient from astrocytes to neurons, concentration of lactate being higher in astrocytes (considered as a reservoir) than in neurons.

Oxidative metabolism of lactate via the TCA cycle is only possible in the presence of adequate concentration of molecular oxygen. The reason is that the TCA cycle is coupled to the mitochondrial electron transport chain (ETC), and the ETC requires molecular oxygen to act as its terminal electron acceptor. The TCA cycle itself does not use molecular oxygen; it utilizes NAD^+^ and FADH as biochemical oxidizing agents that thereby become biochemically reduced to form NADH and FADH_2_. Operation of the ETC is essential to recycle NADH to recreate NAD^+^, and to recycle FADH_2_ to recreate FADH, for the TCA cycle to continue to operate. Without molecular oxygen, the ETC cannot function properly and therefore neither can the TCA cycle.

Of further note is that not all of our patients showed evidence of lactate oxidative metabolism via the TCA cycle, evidenced by no ^13^C fractional enrichment in glutamine detectable above background in four subjects: three of the patients with TBI and one of the “normal” (non-TBI) brain patients (Fig. 4 and Supplementary Table 2). In all cases, we have presented fractional enrichment (%) values *after* subtraction of the natural abundance background of ^13^C that is 1.1% of all carbon atoms.

All the patients studied had detectable glutamine (Fig. 5A, total glutamine concentration), but the apparent lack of ^13^C fractional enrichment in glutamine above background for the four individuals in question may indicate that lactate is not totally universal as a fuel for brain cells. TBI patients four and six had baseline lactate/pyruvate ratios >25 suggesting possible mitochondrial dysfunction in these patients. TBI patient four, however, had no available PbtO_2_ data. In TBI patient six, PbtO_2_ was adequate (23.9 mm Hg) during 3-^13^C lactate perfusion, supporting the theory that mitochondrial dysfunction was the reason for this patient's lack of ^13^C enrichment in glutamine.

Within the limitations of the small number of patients, there were no other obvious distinguishing clinical features for these nonresponders versus those who showed ^13^C enrichment in glutamine. Interestingly, TBI patient seven, a nonresponder, had a baseline lactate/pyruvate ratio median of 22.89 (IQR 22.48–23.76) and PbtO_2_ well above 20 mm Hg both at baseline and during 3-^13^C lactate perfusion. Whether mitochondrial dysfunction can occur despite the lactate/pyruvate ratio being somewhat below the conventional threshold of 25 is an unanswered question.

A limitation of our study is that because its invasive nature, there are no truly normal controls for cerebral microdialysis in humans. In the present study, and in previous studies,^23,24^ we have been able to perform microdialysis in radiologically normal brain areas in non-TBI patients with benign tumors. In this context, this is the closest to a normal control that can be achieved in human studies. Patient considerations and practical matters, however, make such sampling rare. Thus, it was not possible to achieve age- and sex-matching between non-TBI brain controls and TBI subjects. Also, in the present study, for the non-TBI brain controls, there was not enough time to perform an unsupplemented microdialysis period, because there was only sufficient time for microdialysis perfusion with 3-^13^C lactate. Last, while we cannot entirely rule out the possibility that surgery itself may constitute some degree of trauma, we believe this is minor compared with the TBI cohort, given the good clinical state of the postoperative non-TBI patients in comparison with the comatose TBI cohort in neurointensive care.

The issue of lesion progression is outside the scope of this study, because it would have required dedicated imaging (e.g., magnetic resonance imaging) before and after lactate delivery by microdialysis. Our microdialysis catheters were not close to focal TBI lesions (see Methods and Results sections). The rise in lactate/pyruvate ratio (seen with 3-^13^C lactate perfusion, compared with an unsupplemented perfusion period) was considered unlikely to be linked to lesion progression, or vice versa, because the ^13^C labeling showed that oxidative metabolism, evidenced by labeling in the TCA cycle spinout product glutamine, was statistically not significantly different in TBI brain and non-TBI control brain.

### Effect of lactate on microdialysate markers and PbtO_2_

Administration of lactate to the extracellular space resulted in significant increases in microdialysate pyruvate concentrations measured on the ISCUS analyzer, which measures total levels regardless of labeling. ISCUS measurements also showed, albeit without statistical significance, an increase in glucose, consistent with the idea of a glucose-sparing effect of lactate as a brain fuel.^18^

The increase in pyruvate concentrations is as expected through the action of lactate dehydrogenase. An alternative explanation for the increase in pyruvate is that lactate causes increased delivery of glucose to the cells with a subsequent increase in glycolytic pyruvate. Lactate is vasoactive and has been demonstrated both experimentally and in the human brain to increase cerebral blood flow (CBF), mostly likely mediated via prostaglandins.^41–43^ Hence, microdialysis perfusion with lactate might result in the increased local delivery of glucose because of regional increases in CBF. This might also be expected to increase PbtO_2_, which we observed in two of four of the patients for whom we had complete PbtO_2_ data. We believe, however, that the increase in pyruvate concentrations is more likely to be a direct effect through lactate dehydrogenase rather than a more circuitous mechanism through vascular reactivity. This hypothesis (effect through lactate dehydrogenase) is supported by the results of Quintard and colleagues.^19^

### PDH versus pyruvate carboxylation

The ^13^C labeling of glutamine C4 indicates metabolism of 3-^13^C lactate, by way of 3-^13^C pyruvate entry to the TCA cycle through PDH (via 2-^13^C acetyl CoA) and synthesis of α-ketoglutarate during the first turn that spins out to result in glutamine (Fig. 1). PDH is part of the pyruvate dehydrogenase complex (PDC), the rate-limiting step for pyruvate entry into the TCA cycle.^44^ All components of the PDC are expressed in both neurons and astrocytes in culture, but PDC activity is limited in astrocytes through phosphorylation of the PDH alpha subunit (PDHα).^44^ In contrast, neuronal PDC operates at near-maximal levels with much lower levels of phosphorylated PDHα.^44^ Dephosphorylation of astrocytic PDHα increases PDC activity.^44^ Metabolism of astrocytes and neurons thus appears flexible.^44^

In the present study, glutamine C4 exhibited the strongest ^13^C enrichment of the glutamine isotopomers, indicating that overall PDH was the predominant route for 3-^13^C lactate metabolism in the brain, both TBI and “normal” (non-TBI) control (Fig. 4). Smaller ^13^C fractional enrichments were seen in glutamine C2 and C3, consistent with the effect of the second TCA cycle turn after PDH entry that produces a 1:1 mixture of 2-^13^C glutamine and 3-^13^C glutamine, and the first turn of the TCA cycle after PC entry that produces 2-^13^C glutamine (Fig. 4). Accordingly, ^13^C fractional enrichment in glutamine C2 ≥ C3 in those control brain cases showed enrichment in either or both of these positions. Unexpectedly, two of the TBI cases showed ^13^C fractional enrichment at glutamine C3 > C2 (see below for possible explanation). Overall in TBI, fractional enrichments at glutamine C2 and C3 were not statistically different from each other (Wilcoxon signed rank test *p* = 0.6, Student paired *t* test *p* = 0.5). Also, as expected, glutamine C4 fractional enrichment in patients with TBI was significantly higher than at glutamine C3 (Wilcoxon signed rank test *p* = 0.0464; Student paired *t* test p = 0.0484).

PC is a key enzyme for anaplerosis (topping up) of the TCA cycle.^45^ PC is a mitochondrial enzyme regarded as glial rather than neuronal.^45^ Because our microdialysis catheters were inserted into white matter, they are likely to have encountered predominantly glia. While lactate is regarded as a predominantly neuronal substrate, glia can also oxidatively metabolize lactate to a lesser degree.^46,47^

Another potential anaplerotic route for 3-^13^C pyruvate into the TCA cycle is via malic enzyme,^45^ found in both neurons and glia. This would result, on first turn spinout, in a mixture of 2-^13^C glutamine and 3-^13^C glutamine, the same pattern that would form on the second turn if 3-^13^C pyruvate had entered the TCA cycle via PDH. Malic enzyme can work reversibly.^45^ Previously, after administration of 2,3-^13^C_2_ succinate by cerebral microdialysis in patients with TBI, we found spinout of 2,3-^13^C_2_ lactate from the TCA cycle, consistent with the action of malic enzyme.^24^ Whether malic enzyme constitutes a significant anaplerotic (input) route into the TCA cycle in the TBI or control brain remains a possibility.

Malic enzyme can also be involved in “scrambling” of ^13^C label between pyruvate C3 and C2.^48^ 3-^13^C pyruvate can enter the TCA cycle via PDH and PC giving the established labeling patterns in Fig 1. The 2-^13^C pyruvate entry to the TCA cycle via PDH, however, would give 5-^13^C glutamine (first turn) and loss of ^13^C label (second turn). Moreover, 2-^13^C pyruvate entry to the TCA cycle via PC would result in 3-^13^C glutamine (and not 2-^13^C glutamine). Thus, label scrambling may contribute to the two TBI cases with ^13^C fractional enrichment in glutamine C3 > C2.

### Differences between microdialysis delivery of ^13^C-lactate and intravenous perfusion

The delivery and sampling method is a key difference between this study, by microdialysis, which detected labeling in glutamine, and earlier intravenous ^13^C-lactate perfusion studies (see Introduction), which found labeling in glutamate. Moreover, intravenous ^13^C-lactate perfusion studies assessed metabolite labeling patterns in brain tissue, dominated by intracellular species, whereas microdialysis samples extracellular molecules. While glutamate is abundant intracellularly in brain tissue (ca. 5–15 mmol/kg), its concentration is typically low (ca. 1–20 μmol/L) in the extracellular compartment of the brain, in which glutamine dominates (ca. 500 μmol/L).^49,50^

Glutamate is synthesized from the TCA cycle intermediate alpha-ketoglutarate. Glutamate and glutamine are enzymatically interconvertible; the mechanism involves glutamate-glutamine cycling (trafficking) between neurons and astrocytes.^11,49^ Thus, detection of the ^13^C label in glutamate and/or glutamine are hallmarks of TCA cycle operation. In the present study, the finding of glutamine labeling patterns indicative of both PDH and PC operation is consistent with the microdialysis location and consistent with the fact that both delivery of ^13^C-lactate and the collection of products were respectively to and from the extracellular compartment of white matter.

## Conclusion

We have shown that 3-^13^C lactate administered by microdialysis is metabolized via the TCA cycle in both traumatically injured brain and “normal” (non-TBI) control brain in human patients. Lactate is initially metabolized to pyruvate, before entering the TCA cycle mainly via PDH. Lactate administration leads to elevated microdialysate concentrations of pyruvate. In muscle, evidence of TCA cycle metabolism of lactate is lacking, although lactate is metabolized to pyruvate. Based on the data in this study, the hypothesis is accepted that the human brain, both traumatically injured and non-TBI control, can oxidatively metabolize lactate. We found no evidence, however, that resting muscle can oxidatively metabolize lactate. There was insufficient evidence to differentiate statistically between the TBI and non-TBI control brain for lactate metabolism via the TCA cycle. Nevertheless, the upper range values for ^13^C enrichment in glutamine were higher in the TBI brain than non-TBI control brain, which concur with the idea that in certain TBI cases, lactate oxidative metabolism via the TCA cycle can be an “emergency option” for the injured brain.

## Supplementary Material

Supplemental data
