# Supplementary material for: A Comparison of Oxidative Lactate Metabolism in Traumatically Injured Brain and Control Brain
Source: J Neurotrauma. 2018 Sep 1;35(17):2025–35. doi: 10.1089/neu.2017.5459 (PMC6098406; doi:10.1089/neu.2017.5459)
Supplement: Supplemental data [file Supp_Data.pdf]

Supplementary Material

Supplementary Methods

NMR pulse sequences used in this study (originally published in Jalloh I et al. J. Cereb. Blood Flow Metab. (2015) 35, 111–120; DOI: 10.1038/jcbfm.2014.177, © ISCBFM and reproduced with permission of Sage Publications).

^{13}C and ^{1}H NMR spectra were acquired on a Bruker Avance III HD 500 MHz spectrometer (Bruker BioSpin GmbH, Karlsruhe, Germany) with a dual ^{1}H/^{13}C cryoprobe (CP DUL500C/H, Bruker BioSpin GmbH). ^{13}C and ^{1}H NMR spectra were acquired and processed using the TopSpin software (Bruker GmbH). ^{1}H spectra were acquired using the pulse program noesypr1d, a 1D Nuclear Overhauser Effect Spectroscopy (NOESY) experiment using presaturation to suppress the water signal. Acquisition parameters included 32 scans with a d1 (relaxation delay) of 2 seconds. ^{13}C spectra were acquired using the pulse program zgpg30, which has a 30-degree flip angle on the carbon channel, with a d1 of 3 seconds, using 4,096 (4 k) scans and digitizing 64 K points. Power-gated broadband ^{1}H decoupling is achieved using the 'WALTZ-16' supercycle. The receiver gain is set to a constant value in each experiment. Metabolite signals in the samples were identified by comparison of their chemical shifts (p.p.m., reference DSS) to values from NMR databases (BMRB—Biological Magnetic Resonance Bank, University of Wisconsin; HMDB—Human Metabolome Database, Genome Alberta) and to those of our own standards. To confirm the identity of peaks, in selected cases, ^{1}H–^{13}C heteronuclear single quantum correlation (HSQC) spectra were acquired on standards and patients' microdialysate samples.

Supplementary Table S1. ISCUS Clinical Microdialysis Analyzer Measurements

		Unsupplemented perfusion	*Lactate perfusion*	*Unsupplemented perfusion*	*Lactate perfusion*	*Unsupplemented perfusion*	*Lactate perfusion*	*Unsupplemented perfusion*	*Lactate perfusion*	*Unsupplemented perfusion*	*Lactate perfusion*
Patient i.d.	*Centile*	*Glucose mmol/L*	*Glucose mmol/L*	*Lactate mmol/L*	*Lactate mmol/L*	*Pyruvate μmol/L*	*Pyruvate μmol/L*	*Glutamate μmol/L*	*Glutamate μmol/L*	*L/P ratio*	*L/P ratio*
TBI 2 (ii)	Median	2.44	3.13	1.93	5.19	111.74	191.56	2.29	2.82	18.37	26.82
	75th	2.74	3.44	2.37	5.52	123.59	199.14	2.83	3.80	20.28	30.04
	25th	2.29	2.76	1.83	4.96	94.22	181.56	2.05	1.95	16.63	24.94
TBI 4	Median	0.66	0.64	5.79	10.27	171.51	174.32	1.85	8.04	32.66	59.35
	75th	0.80	0.72	5.94	10.78	188.82	196.54	2.79	11.54	38.17	60.40
	25th	0.59	0.53	5.47	10.13	159.04	167.86	1.39	3.22	30.63	52.25
TBI 6	Median	0.56	0.82	2.56	8.19	101.95	129.47	1.67	1.38	25.37	62.80
	75th	0.64	0.85	2.65	8.35	108.30	136.64	2.61	1.51	26.48	66.72
	25th	0.45	0.73	2.34	8.07	89.59	121.00	1.36	1.28	24.59	59.32
TBI 7	Median	1.54	1.72	4.55	9.15	198.02	256.43	23.87	38.60	22.89	37.00
	75th	1.70	2.18	4.68	9.41	206.47	266.86	26.33	112.92	23.76	38.72
	25th	1.46	1.65	4.22	8.61	182.52	240.97	22.86	35.33	22.48	33.47
TBI 8	Median	4.16	3.40	1.65	4.71	124.57	172.62	6.20	3.71	13.34	27.63
	75th	4.28	3.48	2.01	5.04	134.71	175.30	6.58	4.38	14.95	30.19
	25th	3.89	3.32	1.54	4.58	120.00	163.66	5.21	3.36	12.82	26.02
TBI 9	Median	1.21	1.31	2.05	8.05	84.04	123.34	7.79	3.41	24.65	65.72
	75th	1.38	1.50	2.36	8.21	94.18	136.87	9.17	4.16	25.60	88.24
	25th	1.09	1.02	1.93	7.82	78.69	103.50	6.77	3.07	23.61	58.03

TBI, traumatic brain injury; L/P ratio, lactate/pyruvate ratio.

Results are medians, 75th and 25th centiles, during 24 h unsupplemented microdialysis perfusion (with plain central nervous system perfusion fluid) and during 24 h perfusion with 3-^{13}C lactate (sodium salt; 8 millimol/L).

These results are graphed in Figure 2.

Supplementary Table S2. ^{13}C Fractional Enrichment in Glutamine Isotopomers

Patient i.d.	*C4 FE %*	*C3 FE %*	*C2 FE %*
TBI 1 (RF)	11.1	5.3	8.8
TBI 1 (LC)	4.7	5.0	2.7
TBI 2 (i)	13.7	0	0
TBI 2 (ii)	9.5	0	5.7
TBI 3	0	0	3.4
TBI 4	0	0	0
TBI 5	5.5	3.2	3.1
TBI 6	0	0	0
TBI 7	0	0	0
TBI 8	15.8	11.9	7.5
CB 1	8.2	0	0
CB 2	6.8	2.9	3.4
CB 3	5.7	0	1.8
CB 4	0	0	0
CB 5	4.6	0	3.6

TBI, traumatic brain injury; CB, control (non-TBI) ''normal'' brain; RF, right frontal microdialysis catheter; LC, left craniotomy microdialysis catheter; (i), first period of microdialysis; (ii) second period of microdialysis; FE %, ^{13}C fractional enrichment %, expressed after subtraction of natural abundance ^{13}C background; C4, C3, and C2 are the respective carbon atom positions within glutamine (see Fig. 1 for schematic of ^{13}C-labeling). These results are graphed in Figure 4. Footnote: All the above enrichments in glutamine were in the form of single labeling. Double-labeling was not detected in glutamine, evidenced by the absence of ^{13}C nuclear magnetic resonance doublet signals. On the second tricarboxylic acid cycle turn, if a new entering 2-^{13}C acetate molecule joins with labeled oxaloacetate from the first turn to form citrate, this would result in a 50:50 mixture of 2,4 $^{13}C_2$ glutamine and 3,4-$^{13}C_2$ glutamine. The latter molecule if present would show as a doublet signal for glutamine at C3 and a doublet signal for C4. These doublets were absent, however, and the glutamine signals were just singlets. If present, 2,4-$^{13}C_2$ glutamine would give singlets for C2 and C4 because the two ^{13}C atoms are not adjacent, but this labeling pattern is unlikely to have been produced because we did not see any 3,4-$^{13}C_2$ glutamine, which would have accompanied it 50:50. Given that glutamine C4 enrichment was 0–15.8%, then the theoretical maximum enrichment for a doublet would be 2.5% ($= 15.8\% \times 15.8\%$), which would have shown as twin peaks each of half this intensity centered around a C4 singlet, and likewise for C3—but no doublets were seen. For those cases with lower first turn C4 enrichments, the theoretical maximum doublet enrichment would be commensurately lower—e.g., for the lowest (nonzero) enrichment of 4.6% (control patient CB5), the maximum doublet enrichment would have been 0.2% that would have been lost within baseline noise.
